# The development and evaluation of an intervention to promote the uptake of preventive tasks by occupational physicians targeting work-related mental health problems: protocol for the IM-PROmPt-study

**DOI:** 10.1186/s12889-023-16858-3

**Published:** 2023-10-07

**Authors:** S. Orhan Pees, S. H. van Oostrom, F. G. Schaafsma, K. I. Proper

**Affiliations:** 1 National Institute for Public Health and the Environment, Center for Prevention, Lifestyle and Health , Antonie van Leeuwenhoeklaan 9, Bilthoven, 3721 MA The Netherlands; 2 Department Public and Occupational Health, Amsterdam UMC, Amsterdam, The Netherlands

**Keywords:** Prevention, Occupational physicians, Implementation mapping, Peer support groups, Cluster randomized controlled trial

## Abstract

**Objective:**

Work-related mental health problems are a major and growing public and occupational health issue. Although prevention of work-related disease is a central task in the work of occupational physicians, implementation of preventive tasks can still improve. The aim of this paper is to present the development of an intervention to support occupational physicians in the execution of preventive tasks and a protocol for its evaluation.

**Methods:**

An intervention to support occupational physicians has been developed making use of the implementation mapping protocol. The intervention was based on barriers and facilitators for the execution of preventive tasks, input from stakeholders, and evidence-based strategies from literature.

**Results:**

The intervention consists of three peer group supervision meetings directed to preventive tasks. During these meetings, occupational physicians will receive materials and will use goal-setting to formulate their own action plans. The IM-PROmPt-study (Implementation of PReventive tasks by Occupational Physicians) is a two-armed cluster randomized controlled trial, comparing peer group supervision directed to the implementation of preventive tasks for occupational physicians with usual peer group supervision. The evaluation will include an effect and process evaluation to examine if the intervention is successful in supporting OPs to implement preventive activities, specifically aimed to prevent work-related mental health problems.

**Discussion:**

The intervention is expected to lead to more knowledge and awareness of the value of prevention among OPs, anticipated to lead to both organizational and individual gains.

**Trial registration:**

ISRCTN registry; ISRCTN15394765. Registered on 27 June 2023.

**Supplementary Information:**

The online version contains supplementary material available at 10.1186/s12889-023-16858-3.

## Introduction

Work-related mental health problems are a major and growing public and occupational health issue. The proportion of workers in the European Union reporting exposure to risk factors that could negatively affect their mental health increased from 25.0% to 2007 to 44.6% in 2020 [1]. Moreover, in 2020, 18.6% of all reported work-related health problems in the EU were due to mental health problems [2]. These work-related mental health problems are associated with a great deal of personal suffering and loss of quality of life [3]. Moreover, it results in lost productivity, work absence and increased staff turnover, and long-term sick leave, leading to substantial costs for both employers and society [3].

In a study on sick leave data in the Netherlands, it was found that one episode of sick leave due to stress-related complaints lasted on average 101 working days. This accounted for on average 19,151 euros on the side of the employer. Burn-out had the highest number of lost working days and associated costs, with on average 163 working days and associated costs of 30,770 euros [4]. Both the high prevalence and the impact for society and employers highlight the urgency to improve the prevention of work-related mental health problems in the workplace.

Many governments and institutions have developed policies, laws, and guidelines to prevent work-related (mental) health problems of the working population. In the Netherlands, the Dutch Working Conditions Act ensures that all workers are able to work healthy and safely. In the amended Working Conditions Act, prevention of disease and sick leave became an even more central issue as of July 1st, 2017. Among others, a basis contract was introduced. This contains the rights and obligations for employers, employees, and occupational health professionals, and states the requirements for the contracts between occupational health and safety services (OHS) and employers [5–7]. Examples of these rights and obligations are the right of employees to preventively consult an occupational physician (OP), and the right of OPs to freely visit the workplace [5–7].

An evaluation of the revised Dutch Working Conditions Act has shown that whilst more than half of the OPs (62%) indicated that the amended law has allowed them to give more attention to preventive activities, only 17% experienced a reasonable difference and 5% a large difference [6]. Consequently, for many OPs the main focus in their work is still sickness absence and return to work guidance, leaving limited, if at all, time for preventive activities at the workplace [8, 9]. Thus, although prevention at the workplace, and the role of OPs in this, has received more attention in legislation and regulations, the execution in practice still falls short.

To contribute to the prevention of work-related mental health problems in the Netherlands, the aim of this study is to develop strategies to improve the adoption and implementation of preventive tasks in practice by OPs. In this study, the following tasks are defined as being preventive: open consultation hours, workplace visits, preventive medical examinations, periodic occupational health examinations, risk inventory and evaluation, advising about occupational health policy, and consultation with other occupational health professionals. The aim of this paper is twofold: (1) to describe the development of strategies and an intervention for the implementation of preventive tasks by OPs targeting work-related mental health problems and (2) to describe the protocol for a cluster randomized controlled study (RCT) to evaluate the effectiveness of this intervention and the process of implementation.

## Methods

This manuscript is based on the implementation mapping (IM) protocol by Fernandez et al. [10], and describes the development (IM step 1–4) and evaluation (IM step 5) of an intervention to improve the adoption and implementation of preventive tasks by OPs. The IM protocol consists of five steps: (1) conducting a needs and assets assessment and identifying the adopters and implementers; (2) identifying adoption and implementation outcomes, performance objectives and change objectives; (3) selecting theoretical methods and implementation strategies; (4) producing the implementation materials; and (5) evaluating the implementation outcomes [10].

### Step 1 – needs and assets assessment

The aim of the needs and assets assessment was to identify all actors involved in the implementation of preventive tasks by OPs, and to identify barriers and facilitators for the implementation in practice [10]. To identify barriers and facilitators, a non-systematic search in national scientific and grey literature was executed. Only national literature was considered, because of the specific context and occupational health system in the Netherlands. Publications between 2016 and 2021 were included and data extraction was targeted at the barriers and facilitators for the implementation of preventive tasks by OPs. In doing so, a list of barriers and facilitators was created and these factors were grouped by the type (barrier or facilitator); level of implementation (innovation, adopting person, organisation or socio-political context) [11] and actor involved (e.g. OP, employer, employee, occupational health services). The list of barriers and facilitators was ultimately grouped into nine themes and discussed within the research team to find consensus about these clusters.

### Step 2 – implementation outcomes, performance objectives, determinants and change objectives

The aim of the second step was to develop matrices of change objectives with the roles for the different actors, the adoption and implementation outcomes for each of the actors, and the corresponding tasks to achieve these outcomes (i.e. performance objectives) [10]. These matrices can help to understand what needs to change to reach the implementation outcomes. Table 1 shows an overview of the different concepts that are used in the matrices.

**Table 1:** Matrices of change objectives for OPs with an explanation of the used concepts

Implementation outcome	Performance objective	Personal determinant	Change objectives
Key actor and their goal [10].	Tasks required to adopt, implement or maintain the program [10].	Modifiable factors influencing the adoption and implementation (i.e. barriers and facilitators) [10].	Changes needed in the determinant so that performance objective can be achieved [10].

To collect input for the matrices, an interactive session was organized with a group of 19 stakeholders, including OPs, representatives from occupational health services (OHS), representatives from employer and employee organizations, and representatives from several occupational sectors. Before the session took place, the stakeholders were asked to rate the nine themes of barriers as identified in step 1 on importance and changeability on a scale from 0 (unimportant/unchangeable) to 5 (very important/changeable). Facilitators for the implementation of preventive tasks were often the opposite of the barriers. For example, organizing meetings with a multidisciplinary team of (occupational health) professionals could help facilitate the execution of preventive tasks, while a lack of multidisciplinary collaboration could hamper this execution. Therefore, the stakeholders were not asked to score the facilitating factors. During the session, possible solutions and strategies to overcome the barriers for the implementation of preventive tasks by OPs were discussed.

Using the information from this session, implementation outcomes and performance objectives (see Table 1) were developed by the research team. For the three main actors involved in the implementation (OP, employer, employee), implementation outcomes were formulated based on the barriers and facilitators [10]. Consequently, the matrices of change objectives were produced.

### Step 3 – theoretical methods and strategies

Following from the change objectives, the aim of the third step was to select (theory-based or evidence-based) methods to influence the earlier identified determinants, and to develop strategies that are based on these theoretical methods [10]. For this, a second stakeholder meeting was organized with the same group of stakeholders to prioritize the strategies that had been proposed during the first meeting. The stakeholders scored each strategy on the aspects of importance (expected impact) and on feasibility on a scale ranging from 0 (very unimportant/unfeasible) to 5 (very important/feasible). Using idea-prioritization matrices, the strategies and their feasibility were further discussed during the stakeholder meeting.

In addition, the CFIR-ERIC strategy matching tool was used to come up with strategies and methods from literature [12]. This evidence-based tool combines determinants with effective strategies, resulting in a percentage of recommendation for each determinant and strategy [12–14]. Thus, the higher the percentage, the more plausible it would be that a certain strategy would address the determinant. For each of the nine themes of barriers and facilitators found in literature (see step 1) this tool was used to come up with a list of recommended strategies. Next, the strategies with a high percentage of recommendation (> 40%) were further specified by the research team. For this, the taxonomy of behaviour change methods by Kok et al. was used [15].

### Step 4 – implementation protocols and materials

The aim of the fourth step was to produce protocols, activities and materials needed for the implementation of preventive tasks, using the strategies proposed by stakeholders and collected from literature [10]. For the intervention, protocols and materials, the aim was to build upon existing working methods and structures in order to increase its use by OPs. One of these working methods is participating in so-called peer support group meetings (also called peer group supervision or intervision meetings). In the Netherlands, OPs are obliged to participate in these meetings. With their participation, they receive CME accreditation points that are required for maintenance of licensure and certification as OP. However, the topics discussed are to be determined by the peer groups themselves. The peer group meetings take place in fixed groups of approximately eight people. The designed intervention will be introduced in these peer support meetings. Interviews were held with representatives of the Netherlands Society of Occupational Medicine (NVAB) and OPs to make sure the intervention designed would fit to practice and their expectations of the peer-group meetings, and adaptations were made if necessary. In addition, a flyer was developed to use during the meetings. For this flyer, the strategies proposed earlier by the stakeholders and derived from literature (see step 2 and 3) were used.

### Step 5 – evaluation

The last step of IM aims to evaluate the intervention. Therefore, a cluster randomized controlled trial (RCT) will be conducted to evaluate the effectiveness and the process of the intervention. More specific, the following research questions will be addressed:

1. What are the effects of the intervention on the execution of preventive tasks aimed at the prevention of work-related mental health problems and behavioural determinants of OPs?
2. What are the experiences of OPs and other stakeholders with the process of implementation of the intervention?

## Protocol for the IM-PROmPt-study– results from the IM developmental phase

### Step 1 – needs and assets assessment

Based on literature, a list of more than 100 barriers and facilitators for the implementation of preventive tasks by OPs was created and grouped into nine themes: (1) Insufficient knowledge about prevention among employees, employers and OPs; (2) Insufficient multidisciplinary collaboration; (3) (Lack of) knowledge and skills of OP; (4) Costs-benefits of preventive tasks for the employer; (5) Insufficient resources, facilities and time for OP; (6) Familiarity, accessibility and findability of OP among employer and employee; (7) Laws and regulations are not known by employers; (8) Insufficient cooperation between occupational and curative health professionals; (9) Lack of trust in OP because of (financial) dependence on employer. An overview of these themes and an explanation of each can be found in appendix 1.

**Fig. 1:**
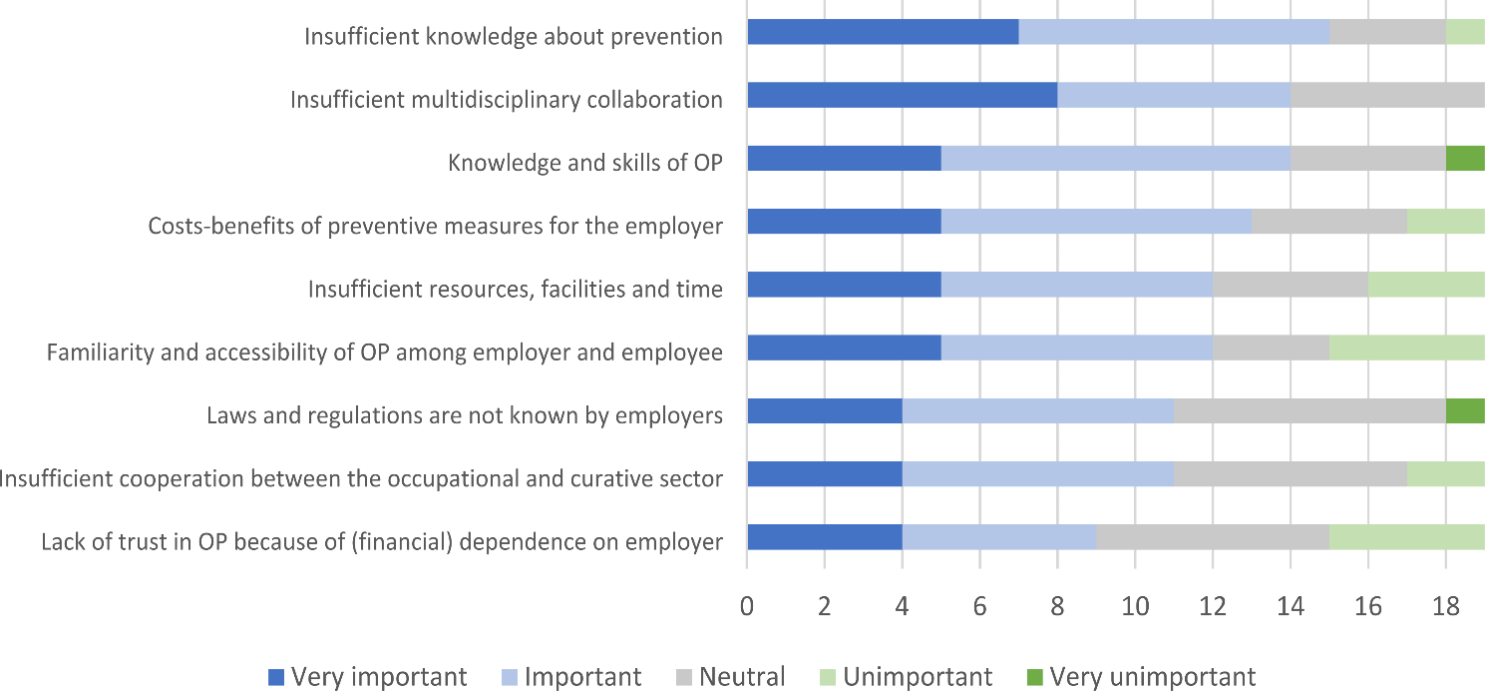
Importance of the 9 themes of barriers for the implementation of preventive tasks by the OP as scored by stakeholders (N = 19). The numbers on the x-axis represent the number of stakeholders who have scored each barrier “very important”, “important”, “neutral”, “unimportant”, or “very important”

**Fig. 2:**
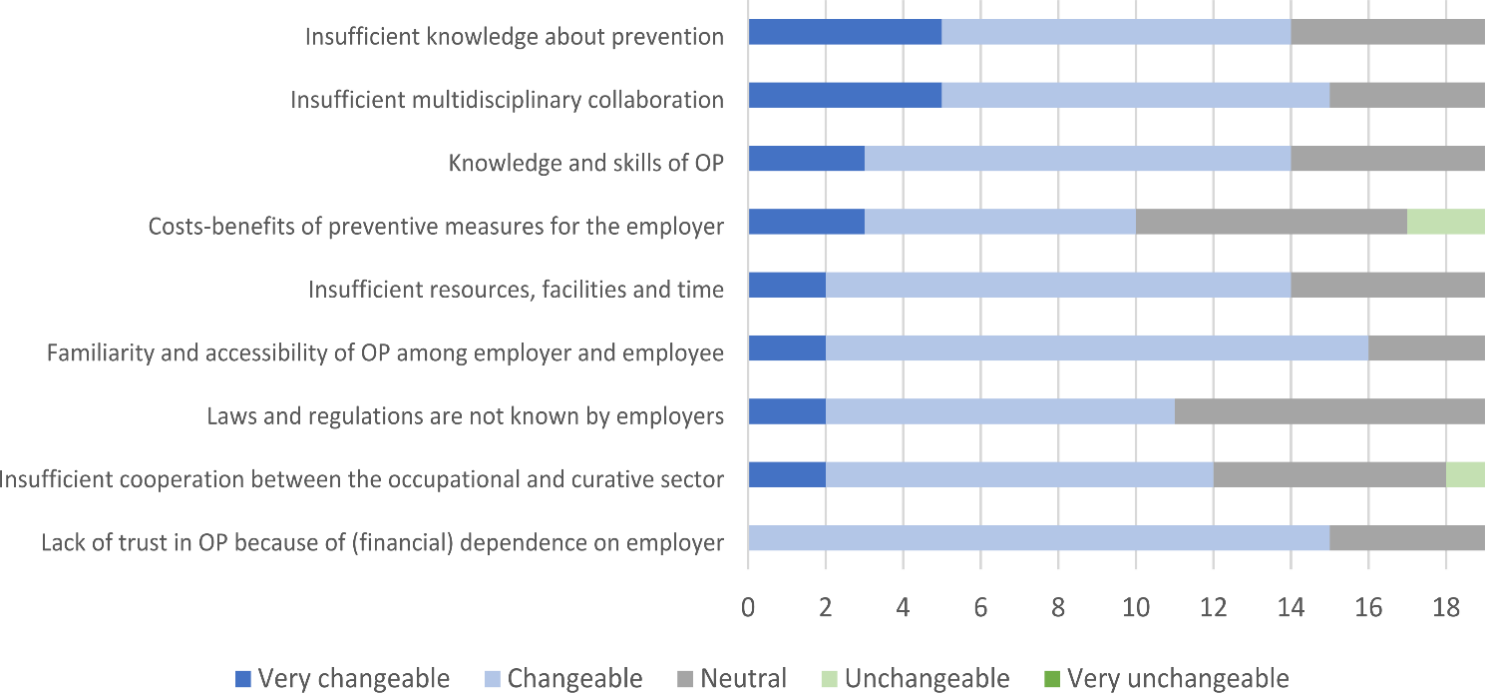
Changeability of the 9 themes of barriers for the implementation of preventive tasks by the OP as scored by stakeholders (N = 19). The numbers on the x-axis represent the number of stakeholders who have scored each barrier “very important”, “important”, “neutral”, “unimportant”, or “very important”

According to the stakeholders consulted, the most important barriers were: insufficient knowledge about prevention among employers and employees; insufficient multidisciplinary collaboration; and lack of knowledge, interest or skills of OPs about preventive tasks (see Fig. 1). At the same time, these were also regarded as some of the most changeable factors (see Fig. 2). Examples of lacking knowledge and skills are lack of knowledge of OPs to organize and carry out specific preventive tasks.

### Step 2 – implementation outcomes, performance objectives, determinants and change objectives

An example of a matrix of change objectives for OPs, including the behavioural outcomes, performance objectives and personal determinants, can be found in Table 2. In this matrix, the behavioural outcome is OPs having sufficient knowledge, skills and interest to carry out their preventive tasks, and to show initiative to put prevention on the agenda of the organization. Related determinants are knowledge, self-efficacy and outcome expectations.

**Table 2:** Matrices of change objectives for occupational physicians, describing the key actor and main goal, tasks required to reach the goal, determinants influencing these tasks (i.e. barriers) and required changes

Behavioural outcome	Performance objective	Personal determinant	Change objectives
OP has sufficient knowledge, skills and interest to carry out preventive tasks and shows initiative and perseverance to put prevention on the agenda within a company	OP integrates the execution of preventive tasks in their other work	Knowledge	Knowledge is shared among OPs through e.g. their own network, magazines, and the professional association.
		Self-efficacy	OP expresses confidence in the ability to use existing materials and protocols in the execution of preventive tasks.

### Step 3 – theoretical methods and strategies

#### Stakeholder input

**Fig. 3:**
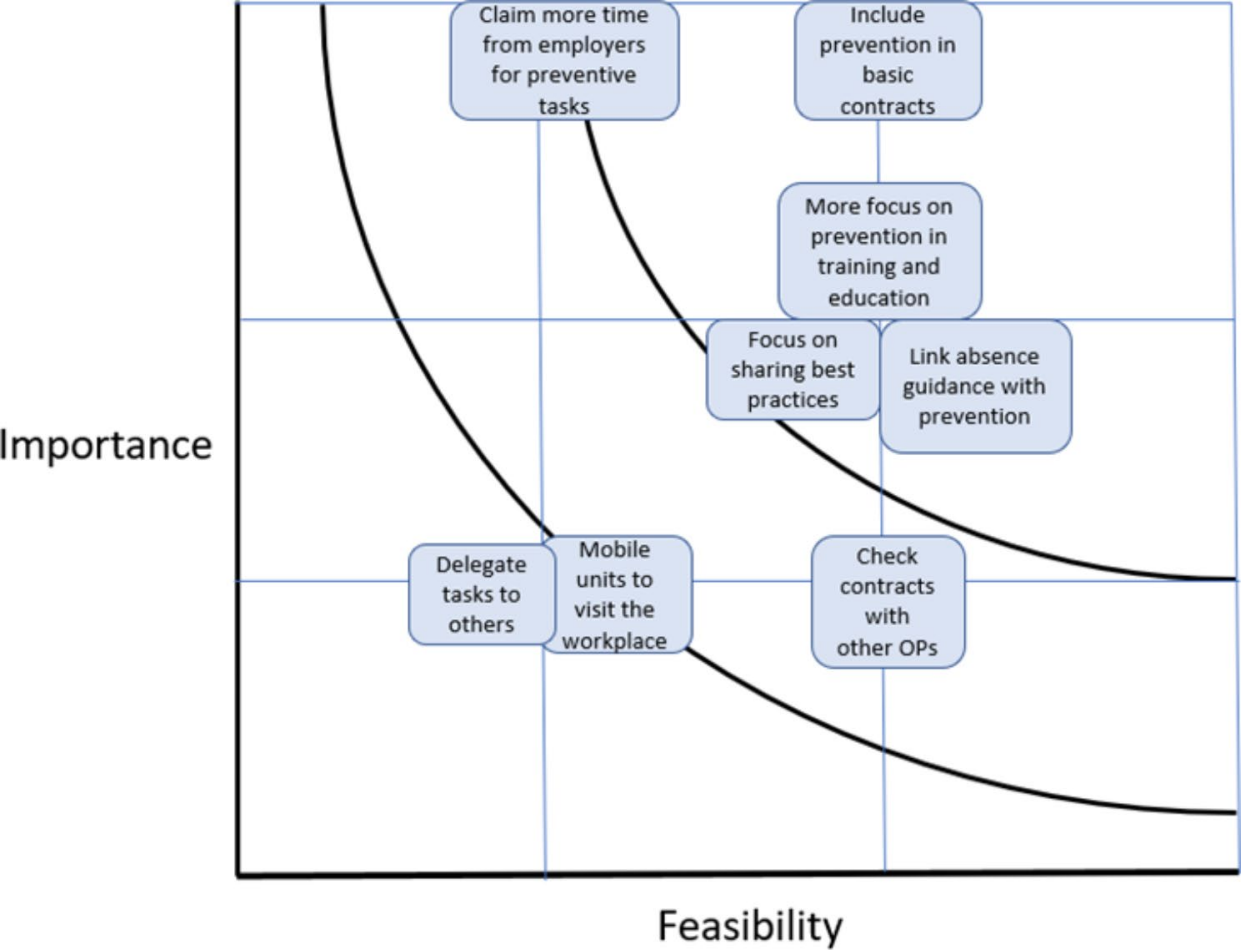
Idea-prioritization matrix with examples of scored strategies

Based on the ratings of the stakeholders as to the importance and feasibility of the earlier proposed strategies, an idea prioritization-matrix was created, see Fig. 3. Strategies located at the right top in Fig. 3 (e.g. include prevention in basic contracts) are the so-called “quick wins” and should be prioritized over strategies placed at the left down (e.g. delegate tasks, mobile units). The latter are both not important and not feasible according to the stakeholders, and should thus be eliminated.

#### Constructs and strategies from literature

After using the CFIR-ERIC matching tool, a list of possible strategies was created from literature to tackle the identified themes of barriers and facilitators. Appendix 2 shows a full list of these ERIC strategies and for which barriers these strategies are helpful to overcome [14].

### Step 4 – implementation protocols and materials

For the intervention, we will use the existing peer group meetings. Within a period of six months, three meetings will be organized with a total of approximately five working hours, in which prevention of mental health problems is the central topic. The chair of each of the intervention groups will be trained and guided in facilitation during the course of the intervention. OPs will be provided with materials, which includes information about work-related mental health problems and ideas about how to incorporate preventive tasks more in their daily practice. Making use of the materials and following different steps, OPs will formulate their own goals with regard to execution of preventive tasks targeting work-related mental health problems to be achieved during the course of the intervention. Advice and input from fellow OPs play an important role in formulating the goals. In short, the peer group supervision groups will focus on 3 important aspects: (1) An interactive introduction into the topic of prevention of mental health problems; (2) Formulating personal goals with input from colleagues; (3) Sharing best practices with regard to prevention. Table 3 shows an overview of the strategies collected in the earlier steps that have been incorporated in this proposed intervention.

**Table 3:** An overview of strategies that have been involved in the intervention. The left column presents strategies proposed by stakeholders, while the right column presents strategies found in literature as being effective

Strategies proposed by stakeholders	Evidence-based strategies from literature [12–14]
Leadership qualities and skills being part of education and training	Conduct educational meetings
Focus on the preventive advisory role and associated knowledge and skills in education and training	Conduct ongoing training
Linking absenteeism guidance to preventive tasks, using existing tools	Make training dynamic
Highlight best practices with regard to prevention	Provide ongoing consultation

### Step 5 – evaluation plan

#### Study design

To evaluate the implementation of the developed intervention, a two-armed cluster randomized controlled trial (RCT) will be conducted. The design of a cluster-RCT has been chosen, because the intervention will be delivered at a group level [16]. The OPs in the groups randomized to the intervention will participate in peer group supervision directed to implementation of preventive tasks targeting work-related mental health problems in their current practice. Groups of OPs randomized to the control condition will not get (initial) access to the intervention and materials. They will be put on a waiting list and participate in their peer group supervision as usual. The evaluation will consist of an effect evaluation and process evaluation. Each individual OP in both the intervention and control group will be asked to complete an online questionnaire at baseline (T0), after 6 months (T1) and after 12 months (T2). In addition, interviews will be held with a selection of participating OPs.

This study will be performed in accordance with the ethical guidelines and regulations laid down in the Declaration of Helsinki. Moreover, this study will be reported in adherence with the CONSORT Guidelines [17]. This study was approved by the Medical Ethics Review Committee of the Academic Medical Center Amsterdam, the Netherlands. The Medical Research Involving Human Subjects Act (WMO) does not apply to the above mentioned study. Written informed consent will be obtained via an online registration form from all participants prior to participating in this study.

#### Study population and recruitment

The study population consists of OPs and will be recruited in two steps. First, we will recruit peer group supervision groups via the Netherlands Society of Occupational Medicine. Second, we will recruit individual OPs within each participating peer group and ask whether they want to participate in the evaluation. Thus, an individual OP can decide not to participate in the evaluation, but could still participate in the specific prevention-oriented peer group supervision meetings. OPs will be excluded from participating in this evaluation when: (1) They have an upcoming retirement or long-term leave (e.g. pregnancy leave) during the follow-up of this study (i.e. 12 months), or (2) They work fewer than 16 h per week as an OP. The latter requirement is to ensure OPs have enough time available to focus on their preventive tasks and change something in their working habits.

#### Sample size and power calculation

The sample size for this study was based on estimating an effect on the primary outcome, the self-reported percentage of time spent on preventive tasks. Using a one-sided Pearson’s chi-squared test of proportions at alpha = 0.05, assuming 0.1 and 0.25 of the total percentage of working hours per week being assigned to prevention tasks in the control and in the treatment groups, respectively, 80 participants were needed in each group to achieve a statistical power of 0.8. Considering a loss to follow-up of 20%, this study would require a sample size of 200 individual participants, of which half in the control and half in the intervention condition. Taking into account that each peer group consists of approximately eight people, this corresponds to approximately 25 peer groups. This analysis was done using the procedure power in SAS, version 9.4, and in consultation with an experienced statistician.

#### Randomization, treatment allocation and blinding

After each individual OP has given their written informed consent for participating in the evaluation and has filled in the baseline questionnaire (T0), randomization will take place at the level of the existing peer groups. Participating groups of OPs will be randomly assigned to (1) the peer group supervision plan directed to the implementation of preventive tasks targeting work-related mental health problems (intervention groups) or (2) the usual peer group supervision condition (control groups). The control groups will be put on a waiting list and receive the peer group supervision materials after the 12 months follow-up of this study. To avoid bias, the randomization process will be executed by two independent researchers. Researcher 1 will assign consecutive numbers to each participating peer group. A computer-generated randomization will then be performed by researcher 2, in order to assign each number to either the intervention or control group. This way, allocation to either one of the groups cannot be influenced. Because of the intervention, blinding for allocation on the level of the participant (OP) is not possible.

#### Effect evaluation

##### Outcome measures

The primary outcome is the execution of preventive tasks aimed at the prevention of work-related mental health problems. This will be assessed by means of the self-reported number of hours spent on each of these tasks, and the self-reported percentage of time spent on prevention, absence and reintegration guidance, and other tasks (e.g. teaching responsibilities). Moreover, OPs will be asked if they would like to spend more time on prevention (ranging from “no, less time” to “yes, considerably more time”). If they want to spend more time on prevention, they will be additionally asked on what specific tasks (e.g. open consultation hour, advising about occupational health policy). Furthermore, questions derived from an existing questionnaire of the Netherlands Society of Occupational Medicine will be used [18]. This questionnaire consists of various statements concerning preventive tasks, whereby OPs must indicate to what extent the statement applies to their situation (ranging from “this applies to a very small degree” to “this always applies” and to what extent they consider it important (ranging from “very unimportant” to “very important”. A selection of 20 statements (out of 58 in the total questionnaire) will be included in the questionnaire. Examples of these statements are: *“I regularly visit the workplace to monitor the interaction between work and health and to evaluate opportunities and risks.”* or *“I give my employers solicited and unsolicited advice about preventive tasks, to prevent and limit health threats”* [18].

Secondary outcomes are the attitude, social influence and self-efficacy of OPs (ASE) [19]; perceived barriers for the execution of preventive tasks; and OPs’ experiences of their work. Their attitude, social influence and self-efficacy will be assessed with the Measurement Instrument for Determinants of Innovations (MIDI), developed by Fleuren et al. [20, 21]. The MIDI consists of 29 determinants, divided based on the different levels of implementation: the innovation, the user (i.e. the OP), the organizational context or the socio-political context [21]. Seven of these determinants will be included in the questionnaire to understand, for example, the degree to which OPs believe they are capable of using the intervention (“self-efficacy”). Perceived barriers will be assessed using the MIDI as well. Five determinants will be included: the formal ratification by the management, sufficient staff capacity, the availability of time, availability of financial resources and availability of material resources and facilities [21]. The first statement is dichotomous, with answer categories being yes and no. The other statements are categorical, with 5 answers ranging from “fully disagree” to “fully agree”. Finally, OPs’ own work experience, such as the work rate and quantity, variety in work and work satisfaction will be determined using the validated Dutch Questionnaire on the Experience and Evaluation of Work (QQEW; Dutch abbreviation: VBBA) [22]. All outcome measures will be determined at baseline, after 6 (T1) and after 12 (T2) months.

##### Covariates

Data on potential confounders will be collected by questionnaire as well. These include both sociodemographic factors and organizational characteristics:

- Individual characteristics of the OPs: age, number of working hours per week, number of years work experience, being self-employed.
- Organizational characteristics: sector, and if they are working for Small and Medium Enterprises (SME) or larger organizations.

##### Data analysis

Data at baseline, 6 months (T1) and 12 months follow-up (T2) will be presented using descriptive statistics. To investigate the effectiveness of the intervention, differences between the intervention and control group in the outcomes at baseline, T1 and T2 will be analysed making use of longitudinal linear or logistic mixed models. If necessary, baseline differences between the intervention and control group will be adjusted for. Consistent with the design of the cluster-RCT and to avoid bias, analyses will be performed following the intention-to-treat principle. This means that all participants randomized will be kept in the groups they were randomized to, regardless of the intervention they received in practice [23]. For the analyses a two-tailed significance level of < 0.05 will be considered statistically significant. All statistical analyses will be performed with SPSS software, version 28.0.1.1.

#### Process evaluation

The process evaluation provides insight into the process of both the implementation of the intervention and the translation of this into their daily practice. We will create a questionnaire for OPs and conduct semi-structured interviews with both OPs and the chair of peer groups assigned to the intervention.

In the questionnaire for OPs, we will use the taxonomy proposed by Proctor et al. (2011) which focuses on eight implementation indicators: acceptability, adoption, appropriateness, feasibility, fidelity, implementation cost, penetration and sustainability [24]. *Acceptability* refers to the degree to which OPs perceive the intervention as agreeable or satisfactory. This will be included in the questionnaire by asking what participants liked and did not like about the peer support group meetings. The a*doption* is simply defined as the uptake or intention to use the intervention. This will be assessed by asking why someone decided to participate in this study and if participation did meet their expectations. *Appropriateness* is defined as the perceived relevance or fit of the intervention to address the issue of lacking implementation of preventive tasks. *Costs* are the costs associated with the implementation effort, including costs of materials and average hourly wage of OPs. For this, administrative data will be used. *Feasibility* is about the extent to which the intervention can be successfully used within, for example if it was feasible to work on one’s personal goals. *Fidelity* measures the degree to which the intervention implemented as intended [24]. This includes the following questions: was the intervention carried out as intended, were all participating OPs present during the peer group meetings and did they actively participate, did OPs formulate achievable goals? *Penetration* is defined as the integration of a practice, but can also be defined as the reach, i.e. the number, proportion and representativeness of participants [25, 26]. Finally, s*ustainability* is the extent to which the execution of preventive tasks is institutionalized within the organisation [24].

In addition, we will link the process and effects to determine if better implementation of the intervention within the peer groups leads to better outcomes with regard to prevention after 12 months. In the effect evaluation, OPs will be asked if they were successful in implementing the preventive tasks and reaching their formulated goals. We aim to determine if OPs in peer groups with higher fidelity have better effects and spend more time on preventive tasks. This will be done making use of sub-group analyses.

Furthermore, during the interviews OPs will be asked for their reasons for using the implementation plan/strategies, how they used it, satisfaction, barriers and facilitators for the implementation of preventive tasks, and intentions with regards to continuation of the preventive tasks [19]. The interviews will be audio recorded and transcribed verbatim. Coding and analyses will be performed using MAXQDA software.

## Discussion

Although preventive tasks should be a central part of the work of OPs in the Netherlands, implementation of preventive tasks is insufficient. To improve the uptake of preventive tasks by OPs, insight is needed into the barriers and facilitators for the utilization of preventive tasks, and into strategies how to overcome these barriers. Making use of literature and stakeholder opinion, and following the Implementation Mapping protocol, we developed an intervention to stimulate OPs to implement preventive tasks in their current practices. The evaluation phase will include an effect evaluation and process evaluation. The aim of this evaluation phase is to examine if this intervention is successful in supporting OPs to implement preventive activities, specifically aimed to prevent work-related mental health problems.

### Relevance and expected impact

More knowledge and awareness of the value of prevention among OPs can ultimately also lead to more knowledge and awareness among employers and employees about work-related mental health problems and preventive measures. It is therefore anticipated that better implementation of preventive tasks by OPs will lead to reduced numbers of work-related mental health problems and to both organizational and individual gains. For OPs, better execution of preventive tasks might not only make the work more varied and attractive, but may also lead to more job satisfaction. For employees, more prevention might contribute to increased awareness, increased job satisfaction and improved workplace culture [27]. Since negative job attitudes and high job demands are found to be predictors of burn-out, increased job satisfaction and improved workplace culture are expected to ultimately result in lower number of burn-out related complaints and reduced health care and sick leave costs [28]. For the organization or employer benefits are, among others, increased work productivity, reduced absenteeism or presenteeism, improved workplace culture and company image [27]. Since episodes of sick-leave due to stress complaints have a long duration and high associated costs (101 working days and 19,151 euros), preventing sick leave can avoid high costs for the employer [4].

If the intervention described in this paper has proven to be effective in supporting OPs in their preventive tasks, widespread implementation of the intervention is anticipated. For this, an additional implementation and dissemination plan will be developed. Moreover, the form of the intervention (peer group supervision focused on goal setting) may also be applicable to prevention of other work-related health problems or relevant themes other than prevention. The process evaluation can help to understand if and how the intervention can be improved in order to do so.

### Methodological considerations

The chosen study design of a cluster randomized controlled trial is a suitable design since the intervention will be delivered at group level [16]. However, one limitation of this design is that participants within a cluster are more likely to resemble each other, and their results can therefore not be seen as independent [23]. In addition, cluster-RCTs have a higher risk of selection and dilution bias than individual RCTs [29]. These methodological limitations have been accounted for in this study by identifying the participants and obtaining their informed consent prior to cluster allocation [29]. This is possible, because existing peer support groups are recruited in this study. A limitation of the designed intervention is that it cannot tackle all identified barriers for the execution of preventive tasks. Some barriers are contextual factors, and related to the organization and financing of the occupational health care system in the Netherlands. These contextual barriers are not easy to solve within the current legislation and regulations. This study is thus conducted within the context of the current system.

Besides these limitations, this study also has some advantages. So far, most research has been limited to only the identification of barriers and facilitators to the implementation of preventive tasks by the OP. Research into the development and evaluation of strategies or interventions that tackle these factors is lacking. Moreover, most studies on the role of the OP focus on absence and reintegration guidance, and not on stimulating the preventive role of OPs. This study fills this research gap. An advantage of using the IM protocol is that we systematically work towards the development of an intervention to support OPs in the execution of their preventive tasks. Another strength is the use of both knowledge from research and literature, as well as practical knowledge from the stakeholders involved. In addition, the strategies used in the intervention are evidence-based and translated to the Dutch context and context of this research. In doing so, we adhere to the existing structure and method of peer support groups. This way, our implementation protocol is not only scientifically relevant, but also fits to practice. The evaluation of this study will focus on the effectiveness and process. This makes our evaluation more comprehensive and will allow us to learn from the implementation process to eventually improve the intervention, before implementing on a wider scale.

### Electronic supplementary material

Below is the link to the electronic supplementary material.

Supplementary Material 1

## Data Availability

Data sharing is not applicable to this article as no datasets were generated or analyzed during the current study.

## Abbreviations

ASE: Attitude, social influence and self-efficacy
IM: Implementation Mapping
IM-PROmPt-study: Implementation of PReventive tasks by Occupational Physicians Study
MIDI: Measurement Instrument for Determinants of Innovation
OHS: Occupational health and safety services
OP(s): Occupational physician(s)
QQEW: Questionnaire on the Experience and Evaluation of Work
SME: Small and Medium Enterprises
RCT: Randomized Controlled Trial
