# Supplementary material for: The development and evaluation of an intervention to promote the uptake of preventive tasks by occupational physicians targeting work-related mental health problems: protocol for the IM-PROmPt-study
Source: BMC Public Health. 2023 Oct 7;23:1948. doi: 10.1186/s12889-023-16858-3 (PMC10560407; doi:10.1186/s12889-023-16858-3)
Supplement: Supplementary file 1 — Supplementary Material 1 [file 12889_2023_16858_MOESM1_ESM.docx]

# Appendices

*Appendix 1: Themes of barriers and facilitators found in literature.*

| *Theme of barriers/facilitators* | *Explanation* |
| --- | --- |
| Insufficient knowledge about prevention among employees, employers and OPs | Includes the barrier of health and safety services being focused mainly on absenteeism and to a lesser extent on prevention, which makes cost savings through prevention not clearly visible. In addition, employers and employees may be insufficiently aware of the risks of occupational diseases, as a result of which they see no added value in prevention, or they may not be able to form a picture of what preventive tasks (such as holding an open consultation hour) entail. |
| Insufficient multidisciplinary collaboration | Includes the OP not cooperating enough with other stakeholders involved in occupational health and prevention, such as the prevention officer, health and safety stakeholders, employee participation bodies or working councils. |
| (Lack of) knowledge and skills of occupational physician | Includes the OP’s possible lack of knowledge, skills and interest in carrying out specific preventive tasks, or the lack of initiative or perseverance to put prevention on the agenda within a company. |
| Costs-benefits of preventive tasks for the employer | Includes factors such as employers being insufficiently confronted with the costs of occupational diseases, which limits investments in prevention. Private resources from employers may also not be sufficient to implement preventive tasks. |
| Insufficient resources, facilities and time for OP | Includes practical limitations for the implementation of prevention, such as a lack of time for the OP (for example due to the shortage of OPs) or the OP not having their own office. Company-specific factors can also play a role, such as language barriers within an international company, or employees who do not come to the office, making a workplace visit difficult to organize. |
| Familiarity, accessibility and findability of occupational physician among employer and employee | Includes that employers and employees do not know the OP, for example due to a high number of changes between OPs, or that the role and tasks of the OP are not known to employers and employees. Moreover, the OP might be difficult to approach in the event of illness or absence. |
| Laws and regulations are not known by employers | Includes that organizations do not have a basic contract with OHS or that preventive tasks by the OP are not included in this contract. Moreover, laws and regulations can also be regarded as complicated, or the possibilities to moving employers to invest in prevention are legally limited. |
| Insufficient cooperation between occupational and curative health professionals | Includes that employees are likely to first report to their general practitioner in case of health complaints. It also relates to insufficient coordination and communication with care providers in curative care (including general practitioners and medical specialists). |
| Lack of trust in OP because of (financial) dependence on employer. | The OP could be (financially) dependent on the employer’s permission for the execution of preventive tasks. For this reason, employees may have no trust in OPs. |

*Appendix 2: ERIC strategies and related barriers and facilitators.*

| ERIC Strategy | Description by Powell et al. (2015) (1) | Theme of barriers/facilitators |
| --- | --- | --- |
| Identify and prepare champions | Hold meetings targeted toward different stakeholder groups (e.g., providers, administrators, other organizational stakeholders, and community, patient/consumer, and family stakeholders) to teach them about the clinical innovation | - Insufficient knowledge about the value of prevention among employees, employers and OPs - Insufficient multidisciplinary collaboration - (Lack of) knowledge and skills of OP about prevention and preventive tasks - Costs-benefits of preventive tasks for the employer - Familiarity, accessibility and findability of OP for employer and employee |
| Conduct educational meetings | Hold meetings targeted toward different stakeholder groups (e.g., providers, administrators, other organizational stakeholders, and community, patient/consumer, and family stakeholders) to teach them about the clinical innovation | - Insufficient knowledge about prevention among employees, employers and OPs - (Lack of) knowledge and skills of OP about prevention and preventive tasks - Familiarity, accessibility and findability of OP among employer and employee |
| Conduct local consensus discussions | Include local providers and other stakeholders in discussions that address whether the chosen problem is important and whether the clinical innovation to address it is appropriate | - Insufficient knowledge about prevention among employees, employers and OPs - Insufficient multidisciplinary collaboration - Insufficient resources, facilities and time for OP |
| Organize clinician implementation team meetings | Develop and support teams of clinicians who are implementing the innovation and give them protected time to reflect on the implementation effort, share lessons learned, and support one another’s learning | - Insufficient multidisciplinary collaboration - Familiarity, accessibility and findability of OP among employer and employee |
| Make training dynamic | Vary the information delivery methods to cater to different learning styles and work contexts, and shape the training in the innovation to be interactive | - Insufficient knowledge about prevention among employees, employers and OPs - (Lack of) knowledge and skills of OP about prevention and preventive tasks |
| Conduct ongoing training | Plan for and conduct training in the clinical innovation in an ongoing way | - (Lack of) knowledge and skills of OP about prevention and preventive tasks |
| Promote adaptability | Identify the ways a clinical innovation can be tailored to meet local needs and clarify which elements of the innovation must be maintained to preserve fidelity | - Insufficient resources, facilities and time for OP |
| Build a coalition | Recruit and cultivate relationships with partners in the implementation effort | - Insufficient multidisciplinary collaboration |
| Identify early adopters | Identify early adopters at the local site to learn from their experiences with the practice innovation | - (Lack of) knowledge and skills of OP about prevention and preventive tasks |
| Inform local opinion leaders | Inform providers identified by colleagues as opinion leaders or “educationally influential” about the clinical innovation in the hopes that they will influence colleagues to adopt it | - (Lack of) knowledge and skills of OP about prevention and preventive tasks |
| Provide ongoing consultation | Provide ongoing consultation with one or more stakeholders in the strategies used to support implementing the innovation | - (Lack of) knowledge and skills of OP about prevention and preventive tasks |
| Conduct local needs assessment | Collect and analyze data related to the need for the innovation | - Insufficient knowledge about prevention among employees, employers and OPs |
